# A survey of the effectiveness of centralized consilia in providing advice on drug-resistant TB

**DOI:** 10.5588/ijtldopen.24.0228

**Published:** 2024-07-01

**Authors:** A. Vasquez, C. Mitnick, A.U. Nyaruhirira, C-Y. Chiang, C.R. Horsburgh

**Affiliations:** ^1^ Department of Global Health, Boston University School of Public Health, Boston, MA, USA;; ^2^ Department of Global Health & Social Medicine, Harvard Medical School, Boston, MA, USA;; ^3^ Management Sciences for Health, Center for Pharmaceutical Management, Pretoria, South Africa;; ^4^ Department of Internal Medicine, Taipei Municipal Wan-Fang Hospital, Taipei, Taiwan;; ^5^ Departments of Epidemiology and Biostatistics, Boston University School of Public Health, Boston, MA, USA

**Keywords:** tuberculosis, multi-drug resistant tuberculosis, MDR-TB, rifampicin-resistant TB, RR-TB, consilia

Dear Editor,

Annually, approximately 410,000 people globally acquire multidrug-resistant TB (MDR-TB) and rifampicin-resistant TB (RR-TB).^1^ However, in 2021, only 161,746 people were enrolled in MDR/RR-TB treatment, with most of the enrollees being adults.^2^ The biggest barrier to care is failure to diagnose drug-resistant TB (DR-TB), but once it is diagnosed, access to treatment and lack of provider experience are important additional barriers.^1^ To expand treatment capacity, centralized hubs of expertise have been set up in many countries to provide either advice and/or direction in selecting treatment regimens and monitoring for tolerability and effectiveness. However, centralization of care may also be a barrier to prompt treatment initiation, especially when centralized decision-making is required before drugs are released. These systems, called ‘consilia’, evolved when MDR/RR-TB treatment was first introduced, and control was prioritized over access.^3^ Currently, WHO provides guidelines on the duration and composition of treatment, but consilia may still be important in some settings for translating these guidelines into individual patient decisions.^4^ In 2000, the first forum for advice on MDR/RR-TB treatment, the ‘Green Light Committee (GLC)’ was created to improve access to quality-assured second-line anti-TB drugs.^3^ In 2014, because many care providers had limited experience in devising and monitoring treatment for DR-TB, WHO recommended that MDR-TB and extensively drug-resistant TB (XDR-TB) case management be overseen by centralized teams at regional levels. These are meant to help with the discussion of difficult cases and serve as the ‘gateway to accessing second-line drugs and/or the new drugs’.^4^ A typical TB consilium is made up of clinicians, public health officers, microbiologists, pharmacologists and other relevant roles. Although consilia aim to improve care for MDR-TB and XDR TB, there are both advantages and disadvantages. Advantages include the ability to receive expert advice, collaboration amongst TB experts, peer quality assurance and potential teaching opportunities. Disadvantages include a slower response rate (potentially leading to delays in initiating treatment) and a limited number of cases that can be discussed.^5^ RESIST-TB and the Union’s DR-TB Working Group sought to examine the role of centralized decision-making as perceived by its users and to determine how it might be improved. We therefore performed a survey examining MDR/RR-TB decision-making and recommendations within different countries.

A questionnaire with 17 questions was sent by e-mail to approximately 1,500 members of the Tuberculosis Section of the International Union Against Tuberculosis and Lung Disease (The Union) – see Supplementary Data for the questionnaire. Members of The Union were also encouraged to share the survey with non-members. The survey was open from March to June 2023. We excluded responses that answered which country they were responding from but did not complete any subsequent questions. Responses were stratified by country. In addition, we grouped the 38 free-response recommendations by categories based on keywords to gain insight into needs and potential improvements in MDR/RR-TB care support.

We received 175 survey responses from 46 countries, but 70 respondents did not initiate the questionnaire, leaving 105 responses from 46 countries for analysis. Of the 46 countries, 13 (28%) were in the WHO African region, 5 (11%) in the Eastern Mediterranean, 11 (24%) in the European, 6 (13%) in the Americas, 2 (4%) in Southeast Asian and 9 (20%) in the Western Pacific. Of the 46 countries represented in this analysis, 38 (84.4%) had a consilium (or several consilia) that gave advice on MDR/RR-TB treatment regimens and 8 (21.1%) did not. Of the 38 countries with a consilium, 29 (76.3%) had circumstances requiring their advice, whereas 9 (23.7%) did not. Of these 29 countries, 8 were in Africa, 3 in the Eastern Mediterranean, 7 in Europe, 4 in the Americas, 2 in Southeast Asia and 5 in the Western Pacific. There was variability in the circumstances in which consilium advice was required (Figure). Of the consilia in the 29 countries where advice was mandatory, 23 (79%), provided advice within 2 weeks, 4 (14%) within 2–3 weeks and 2 (7%) in four or more weeks. In the open response field, our respondents observed that consilia were often understaffed and lacked adequate financial and social support for patients. They also identified challenges that may have been outside the remit of the consilium that adversely affected patient treatment decisions, such as lack of drug availability and limited access to second-line susceptibility testing. Specifically, in 27% of the countries, respondents identified the lack of access to susceptibility testing for drugs used in MDR/RR-TB treatment as an important barrier. In addition, lack of access to bedaquiline, pretomanid and or delamanid was cited in 12%, and the high cost of treatment to the patient was cited in 7%.

**Figure.**
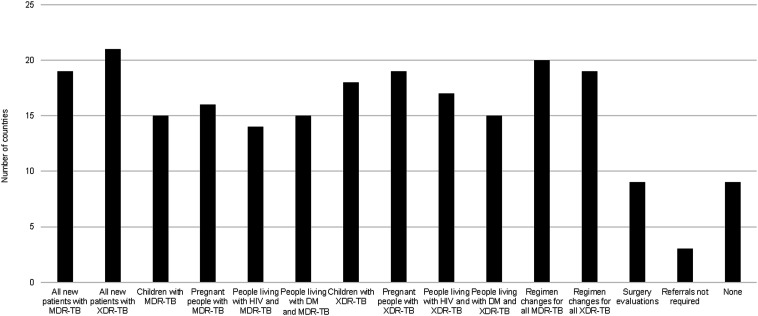
Circumstances when advice from consilium must be sought according to national guidelines. MDR-TB = multidrug-resistant TB; XDR-TB = extensively drug-resistant TB; DM = diabetes mellitus.

These results show substantial variability in the circumstances where consilium input must be sought (per local TB Program guidelines) and considerable differences in the time taken to respond with treatment advice. The results of our survey on consilia are similar to challenges reported in the literature: other studies have reported that shortages of expert staff to manage high caseloads can slow response times;^5^ and a French consilium (comprised of a small number of experts) also noted average response time of one month.^6,7^ In comparison, the European Respiratory Society/WHO consilium (comprised of 54 experts) reported an average response time of 48 hours, and five other countries (four of them in Europe) reported responses in 1–5 days.^7^ The French consilium saw an increase in the availability of the new drugs over time, whereas access and availability remain challenging in the countries responding to our survey. In countries with small numbers of patients with MDR/RR-TB and where clinical expertise is limited, consilia can function to ensure quality of care. An alternative is to set up special treatment centers rather than overseeing inexperienced clinicians treating occasional patients. In countries where the burden is substantial, consilia may need to focus on training providers of MDR/RR-TB care so they can function without oversight. Moreover, where there are substantial numbers of children with MDR/RR-TB, there may be a need for specialized support.

These results demonstrate a substantial degree of variability between countries and the need to improve responsiveness for treatment guidance. The availability of Group A second-line drugs and susceptibility testing to these drugs continues to be suboptimal.^8^ The functions of consilia may need to be more closely matched to the local conditions to reflect the levels of MDR/RR-TB in the population.

## References

[1] World Health Organization. Global tuberculosis report, 2023. Geneva, Switzerland: WHO, 2024.

[2] World Health Organization. Global tuberculosis report, 2022. Geneva, Switzerland: WHO, 2023.

[3] Yassin MA, . Performance-based technical support for drug-resistant TB responses: lessons from the Green Light Committee. Int J Tuberc Lung Dis 2020;24:22–27. 32005303 10.5588/ijtld.19.0376

[4] World Health Organization. Policy implementation package for new TB drug introduction. Geneva, Switzerland: WHO, 2014.

[5] Tiberi S, . Challenging MDR-TB clinical problems - The case for a new Global TB Consilium supporting the compassionate use of new anti-TB drugs. Int J Infect Dis. 2019;80:S68–S72. 10.1016/j.ijid.2019.01.040 30690212

[6] Guglielmetti L, . Multidisciplinary advisory teams to manage multidrug-resistant tuberculosis: the example of the French Consilium. Int J Tuberc Lung Dis. 2019;23(10):1050–1054. 31627768 10.5588/ijtld.18.0779

[7] D'Ambrosio L, . Team approach to manage difficult-to-treat TB cases: Experiences in Europe and beyond. Pulmonology. 2018;24(2):132–141. 29229274 10.1016/j.rppnen.2017.10.005

[8] Tiberi S, . Drug resistant TB - latest developments in epidemiology, diagnostics and management. Int J Infect Dis. 2022;124 Suppl 1:S20–S25. 35342000 10.1016/j.ijid.2022.03.026

